# Associations of Plasma BDNF and *BDNF* Gene Polymorphism with Cardiometabolic Parameters in Thai Children: A Pilot Study

**DOI:** 10.1155/2023/9668626

**Published:** 2023-03-24

**Authors:** Kanjana Suriyaprom, Banchamaphon Pheungruang, Somchai Pooudong, Pumpath Putpadungwipon, Chutima Sirikulchayanonta

**Affiliations:** ^1^Faculty of Medical Technology, Rangsit University, Paholyothin Road, Pathumthani 12000, Thailand; ^2^School of Nutrition and Dietetics, Institute of Public Health, Suranaree University of Technology, Nakhon Ratchasima 30000, Thailand; ^3^Department of Tropical Nutrition and Food Science, Faculty of Tropical Medicine, Mahidol University, Rajvithi Road, Rajthevee, Bangkok 10400, Thailand; ^4^College of Medicine, Rangsit University, Paholyothin Road, Pathumthani 12000, Thailand

## Abstract

**Background:**

Childhood obesity is an important public health crisis worldwide. The brain-derived neurotrophic factor (BDNF) has been demonstrated to play a role in controlling energy homeostasis and cardiovascular regulation.

**Objectives:**

To examine brain-derived neurotrophic factor (BDNF) levels and anthropometric-cardiometabolic and hematological parameters in obese and nonobese children and to determine whether two *BDNF* gene polymorphisms (G196A and C270T) are linked to BDNF levels, obesity, and anthropometric-cardiometabolic and hematological parameters among Thai children.

**Methods:**

This case-control study included an analysis of 469 Thai children: 279 healthy nonobese and 190 obese children. Anthropometric-cardiometabolic and hematological variables and BDNF levels were measured. Genotyping of *BDNF* G196A and C270T was performed using the polymerase chain reaction-restriction fragment length polymorphism technique.

**Results:**

Children in the obese group had significantly higher white blood cell counts and some cardiometabolic parameters. Although the difference in BDNF level between the nonobese and obese groups was not significant, BDNF level was significantly positively correlated with hematological and cardiometabolic parameters, including blood pressure, triglycerides, and triglycerides and the glucose index. The *BDNF* G196A polymorphism in children was only associated with decreased systolic blood pressure (*p* < 0.05), while the *BDNF* C270T polymorphism was found not to be related to BDNF levels, obesity, or other parameters after adjusting for potential covariates.

**Conclusions:**

These findings in Thai children suggest that obesity is associated with increased cardiometabolic risk factors, but not with BDNF levels or the two *BDNF* polymorphisms studied, while the *BDNF* G196A polymorphism is a beneficial marker for controlling blood pressure among Thai children.

## 1. Introduction

Globally, the prevalence of childhood obesity is too high, including in Thailand. Obesity is one of the most serious health problems because it is the increased risk of various diseases, including Type 2 diabetes, cardiovascular disease, hypertension, and fatty liver disease [1]. The pathophysiology of obesity is multifactorial and comprises complex interactions among biological, environmental, behavioral, and genetic factors [1, 2]. The brain-derived neurotrophic factor (BDNF) is a neurotrophin that acts through the tropomyosin-related kinase B (TrkB) receptor and is involved in memory formation, energy homeostasis, appetite, and cardiovascular regulation [3–5]. Previous studies have examined the relationships of circulating BDNF levels with obesity and cardiometabolic parameters [6–10]; however, these studies have conflicting results that are complex to explain. Regarding the relationship between *BDNF* polymorphisms and obesity, the *BDNF* G196A (Val66Met) polymorphism (rs6265) was chosen as one of the two tags in the present study because this common *BDNF* polymorphism affects intracellular packaging of the precursor of BDNF (proBDNF) [11]. However, there are conflicting data regarding the association between the *BDNF* G196A polymorphism and obesity [12–17]. The other polymorphism assessed in the present study, *BDNF* C270T (rs56164415), is in a noncoding region with a promoter function (5′-untranslated region (UTR)), and it may modify the efficacy of BDNF translation and contribute to region-specific quantitative BDNF imbalances in the brain [18]. Furthermore, the information on these two *BDNF* polymorphisms with regard to obesity, cardiometabolic parameters, and plasma BDNF concentrations remains scarce, especially among Thai children. Thus, the purposes of this study were to examine plasma BDNF levels and anthropometric-cardiometabolic and hematological parameters in obese and nonobese Thai children and to determine whether two *BDNF* polymorphisms (G196A and C270T) are linked to their plasma BDNF levels, obesity, and anthropometric-cardiometabolic and hematological parameters.

## 2. Methods

### 2.1. Study Population

The present observational case-control study included 469 Thai children aged 8–12 years from public schools in Bangkok under the Ministry of Education. A total of 190 obese children (125 males, 65 females) and 279 healthy nonobese children (138 males, 141 females) were chosen among children receiving a check-up during a health screening program from June 2019 to January 2021. The sample size of our study was calculated based on a study by Míguez-Burbano et al. [19], which reported a standard deviation (SD) of BDNF levels of 6,419 pg/ml and a true potential difference of BDNF levels of 2,477.1 pg/ml between the compared groups (statistical power: 90%, type I error = 0.05). A physical examination and medical history check were conducted by the same medical doctor for all subjects throughout the study. Subjects with the obesity-associated syndrome; liver, kidney, or cardiovascular diseases; or any inflammatory or infectious diseases and those who used prescription medication for any reason were excluded from this study.

This study was conducted under the principles of the Declaration of Helsinki, and the protocol was approved by the Ethics Committee of Rangsit University (RSEC 12/2560). Prior to data collection, the aims of the study were carefully described to the children and their parents. Whether the study participants and/or their parents were the ones to sign the consent form, all participants agreeing to participate signed a consent form.

### 2.2. Anthropometric Measurements

Weight, height, and waist circumference (WC) were measured for all subjects. Weight was measured to the nearest 100 g using a carefully calibrated beam balance (Detecto®, Cardinal Detecto Scale Manufacturing, USA), and children were instructed to stand on the balance and look straight with light clothing and no footwear. Height measurements were taken three times and measured to the nearest 0.1 cm using a calibrated stadiometer (Microtoise, CMS instruments, London, UK). WC measurement was typically taken three times and measured to the nearest 0.1 cm by trained technicians. The body mass index (BMI) was calculated as weight (kg) per height (m^2^) and transformed into BMI z-scores that were adjusted for gender and age according to the new World Health Organization reference [20]. The cut-off values for weight status were as follows: normal weight BMI z-score ≤1 SD to >−1 SD and obese BMI z-score >2 SD. Blood pressure (BP) measurements were repeated a minimum of three times with two-minute intervals between measurements by a nurse after 5 to 10 minutes of rest in the seated position.

### 2.3. Measurement of Biochemical Parameters

Ten milliliters of blood were collected from each subject in the morning after a 12 h fast between 7.30 a.m. and 9.00 a.m. to minimize the effects of a possible circadian variation. Plasma samples were drawn into tubes containing ethylenediaminetetraacetic acid (EDTA) and immediately placed on ice, then centrifuged at 4°C for 15 min at 1,200 × *g* to obtain the plasma. Immediately after centrifugation, plasma samples were divided into aliquots and stored at −80°C until the assay was performed. Plasma BDNF levels were determined in duplicate using an ELISA protocol according to the manufacturer's instructions (R&D Systems, Europe). The intra-assay and interassay errors were approximately <5% and <9%, respectively. Glucose and lipid profiles (total cholesterol (TC), high-density lipoprotein cholesterol (HDL-C), low-density lipoprotein cholesterol (LDL-C), and triglycerides (TG)) were measured using enzymatic methods on a Cobas Integra 400 Roche analyzer with commercially available kits (Roche Diagnostics GmbH, Mannheim, Germany). Non-HDL-C was derived from TC minus HDL-C. The triglyceride-glucose (TyG) index was calculated using the formula: ln(fasting TG (mg/dL)  × fasting plasma glucose (mg/dL)/2). Whole blood collected in an EDTA tube was used to determine hematological variables, such as white blood cell (WBC), red blood cell (RBC), and platelet counts using the COULTER® Hematology Analyzer (Beckman Coulter, Inc., Indiana, USA).

### 2.4. Polymerase Chain Reaction-Restriction Fragment Length Polymorphism (PCR-RFLP) Technique

Genotyping was performed using the PCR-RFLP technique. The DNA was extracted from peripheral leukocytes in EDTA-treated whole blood using a FlexiGene DNA Kit (Qiagen, Hilden, Germany). Genotypes of the single nucleotide polymorphisms (SNPs) G196A and C270T were amplified using PCR (Applied Biosystems, California, USA). The G196A polymorphic locus was amplified using the forward primer 5′-ATCCGAGGACAAGGTGGC-3′ and the reverse primer 5′-CCTCATGGACATGTTTGCAG-3′. The forward primer 5′-CAGAGGAGCCAGCCCGGTGCG-3′ and the reverse primer 5′- CTCCTGCACCAAGCCCCATTC-3′ were used to amplify the position of *BDNF* C270T. The PCR products were digested overnight with 10 U of *PmlI* or *HinfI* restriction endonuclease for the *BDNF* G196A and C270T polymorphisms, respectively. The digestion products were separated and stained with ethidium bromide according to the protocols previously described by Suriyaprom et al. for the *BDNF* G196A polymorphism [21] and by Kunugi et al. for the *BDNF* C270T polymorphism [22].

### 2.5. Statistical Analysis

Statistical analysis was performed using SPSS Statistics for Windows version 11.5 (SPSS Inc., Chicago, IL, USA). The normal distribution of each continuous variable was tested using the Kolmogorov–Smirnov test. Continuous variables with a normal distribution are expressed as mean ± standard deviation (SD), and those with a nonnormal distribution are presented as the median with an interquartile range (25th–75th percentile). For comparisons between the obese and nonobese groups, the Mann–Whitney *U*-test was used for the parameters with a nonnormal distribution, while the independent samples *t*-test was used for the parameters with a normal distribution. Allele and genotype frequencies were compared between the two groups using the chi-square test. The Hardy-Weinberg equilibrium was assessed using Pearson's *χ*^2^-test. The correlation between bivariate variables was assessed using Pearson's correlation. To assess relationships between obesity as a dependent variable and other potential factors, binary logistic regression was applied. The goodness-of-fit of binary logistic regression models was established using the Hosmer–Lemeshow test. The analysis results were statistically significant at *p* < 0.05.

## 3. Results

A total of 469 children were enrolled in this study, including 190 obese children and 279 nonobese children.  Table 1 compares the anthropometric-cardiometabolic and hematological variables in nonobese and obese children. The difference in plasma BDNF between the nonobese group (32.2 ± 6.4 ng/ml) and the obese group (33.8 ± 6.4 ng/ml) was not significant (*p*=0.079). Regarding the effects of gender differences, obesity, and the interaction between gender and obesity on BDNF levels by using two-way ANOVA, the results revealed that gender (df = 1, *F* = 0.776, *p* > 0.05), obesity (df = 1, *F* = 2.335, *p* > 0.05), and the interaction between gender and obesity (df = 1, *F* = 0.174, *p* > 0.05) did not affect BDNF levels in Thai children. However, higher obesity phenotypes (weight, WC, and BMI z-score) were observed in the obese group compared to the nonobese group (*p* < 0.01). The cardiometabolic parameters, including glucose, TyG index, non-HDL-C, TG, systolic BP, and diastolic BP, were significantly higher, whereas HDL-C level was significantly lower in the obese group than the nonobese group (*p* < 0.01). Furthermore, with the exception of WBC count, the differences in age and hematological parameters between the obese and nonobese groups were not significant. Correlation coefficients of BDNF concentration with other parameters are presented in Table 2. BDNF level was positively correlated with BP, TG, TyG index, and hematological parameters (*p* < 0.05). To evaluate the relationships of gene polymorphisms (*BDNF* G196A and C270T) with anthropometric-cardiometabolic and hematological parameters, we performed logistic regression analysis. When the SNP G196A was used as a dependent variable, the results manifested that decreased systolic BP was associated with the SNP G196A (*p* < 0.05) after adjusting the variable for the covariates gender and age. When the SNP C270T was used as a dependent variable, there were no significant associations of anthropometric-cardiometabolic and hematological parameters with the C270T polymorphism (*p* > 0.05) after adjusting the variable for the covariates; the results are shown in Table 3. The genotypic and allelic frequency results for the SNPs *BDNF* G196A and C270T in the obese and nonobese groups are demonstrated in Table 4. All participants were genotyped for the SNPs *BDNF* G196A and C270T. The genotypic and allelic distributions of these two *BDNF* polymorphisms were not significantly different between the obese and nonobese groups, and the SNPs *BDNF* G196A and C270T were not related to obesity (*p* > 0.05). Moreover, there were no significant deviations from Hardy–Weinberg equilibrium for the SNPs *BDNF* G196A and C270T in nonobese participants (*p*=0.830 and *p*=0.837, respectively) or obese participants (*p*=0.105 and *p*=0.543, respectively). The logistic regression results and ORs for possible associations between obesity and age, gender, BDNF concentration, glucose, TyG index, non-HDL-C, WBC count, and the SNPs *BDNF* G196A and C270T are shown in Table 5. Three variables, TyG index (OR = 3.113, *p*=0.009), glucose (OR = 1.088, *p*=0.005), and WBC count (OR = 1.064, *p* < 0.001), were statistically significantly related to obesity. The Hosmer–Lemeshow goodness-of-fit test (*χ*^2^ = 11.7, *p*=0.189) was not statistically significant; thus, the fit between the predictive model and the data was acceptable.

## 4. Discussion

To the best of our knowledge, our study indicated for the first time that obesity is linked to increased cardiometabolic risk factors but is not associated with plasma BDNF levels or the two *BDNF* polymorphisms studied (G196A and C270T) in Thai children. However, the *BDNF* G196A polymorphism was significantly linked with decreased systolic BP in Thai children.

Our findings in Thai children did not support the concept that BDNF inadequacy induces a metabotropic impairment leading to obesity [3]. The present study showed a lack of association between BDNF levels and obesity and its phenotypes as measured by BMI and WC in Thai children; nevertheless, our findings are consistent with previous reports in a meta-analysis [7] and in studies of Spanish prepubertal children [6]. However, there are also studies with contradictory results. The findings of Katuri et al. revealed that serum BDNF levels were decreased in obese south Indian young adults compared to nonobese [8]. On the other hand, Roth et al. reported that fasting BDNF levels were higher in obese children than in those with a normal weight and were positively related to BMI [9]. Nevertheless, the relationship between BDNF and obesity seems to be complex, and many factors are considered to be involved in this relationship, including biological, environmental, behavioral, and genetic factors [4, 6].

Regarding the *BDNF* polymorphisms, there is insufficient data to determine whether the *BDNF* C270T polymorphism is linked to obesity, BDNF level, and cardiometabolic profile, especially in children. The *BDNF* C270T polymorphism may cause a loss of transcription factor binding, which may alter the transcriptional efficacy [18]. The study on eating disorders found a significant association between the *BDNF* C270T polymorphism and low BMI in individuals with bulimia nervosa [23], whereas our study did not confirm this association. Moreover, we did not detect relationships between the *BDNF* C270T polymorphism and obesity and cardiometabolic profiles in Thai children, which are in line with data from middle-aged Caucasian (Croatian origin) veterans showing a lack of a significant association between this polymorphism and BMI and lipid profile [24]. Nevertheless, contrasting results in the association of the *BDNF* C270T polymorphism with any obesity-related traits, cardiometabolic profiles and BDNF levels could be explained by the different ethnic backgrounds and behaviors of the study participants.

We hypothesized that there would be a higher frequency of the *BDNF* G196A polymorphism among Thai children with low plasma BDNF levels or with obesity, but the present study rejects this hypothesis. Although some studies have demonstrated a role for BDNF in regulating body weight and food intake [3, 4], our findings revealed a lack of a significant association between the *BDNF* G196A polymorphism and obesity and anthropometric traits in Thai children, and these data are in line with case studies of Serbian adolescents [12] and Canadian youth [13]. However, there are also conflicting results. Skledar et al. demonstrated a significant relationship between the presence of the A allele and obesity in Croatian Caucasian children and adolescents [16]. In contrast, studies in German children and adolescents [14] and Chinese children [15] illustrated an association between the A allele and lower adiposity. Therefore, future research should aim to determine not only the associations between the *BDNF* G196A polymorphism and obesity traits as measured by BMI and WC but also the relationships between the carriers of different alleles and body composition and lifestyle factors because obesity is an outcome of multiple factors, including environmental and genetic influences.

Regarding the association between the *BDNF* G196A polymorphism and BDNF levels, results from previous studies are contradictory [11, 25]. However, the present study rejected the hypothesis that the *BDNF* G196A polymorphism has a direct effect on circulating BDNF because plasma BDNF levels were unrelated to the *BDNF* G196A polymorphism in Thai children. Our finding is in line with those of other large studies that have found no association between BDNF levels and the *BDNF* G196A polymorphism in a meta-analysis of published studies [25]. Furthermore, Terracciano et al. found other variants in the *BDNF* gene (rs7170215 and rs11073742) that map within and near the *NTRK3* gene, which encodes a tyrosine kinase receptor that might regulate the level of serum BDNF by playing a role in feedback mechanisms regulating the expression and storage of BDNF [25]. Therefore, future studies should determine the effects of other genetic variants and environmental and physiological factors on circulating BDNF concentrations. However, epigenetic mechanisms and posttranscriptional processes, such as histone acetylation and DNA methylation, should also be considered, as these can affect *BDNF* expression [26].

Previous studies had indicated roles for BDNF in cardiovascular and metabolic regulation [3, 4], and our findings revealed that BDNF level was positively correlated with cardiometabolic risk factors in Thai children. High BP, dyslipidemia, and high WBC levels are well known to be risk factors for cardiovascular diseases. Furthermore, the TyG index is a simple and inexpensive clinical marker reflecting insulin resistance that is associated with high risk of experiencing an atherosclerotic cardiovascular event [27]. However, little is known about the relationship between BDNF and the TyG index. To our knowledge, this is the first study that provides evidence to support a positive correlation between plasma BDNF and the TyG index among Thai children. BDNF is associated with the pathogenesis of cardiovascular disease by the stimulation of atherogenesis and plaque instability via the stimulation of NAD(P)H oxidase and the inflammation after myocardial infarction [5, 28]. Our findings supported the positive relationships between BDNF and WBC count, which is an inflammation marker. Moreover, studies in animal models have indicated roles for BDNF in regulating the balance of angiotensin signaling, with a long-term elevation of BDNF in the paraventricular nucleus of the hypothalamus being associated with increasing BP [29]. Therefore, our findings supported the positive association between BDNF levels and BP, which is in accordance with the previous reports that suggested functions for BDNF in cardiovascular homeostasis and that increased BDNF levels were associated with increased risk of cardiovascular disease [4, 28].

Regarding the *BDNF* polymorphisms, in vitro studies demonstrated that the A variant of the G196A polymorphism is associated with lower activity-dependent BDNF secretion from hippocampal neurons [11]. Shalev et al. also found that *BDNF* G196A can modulate hypothalamic-pituitary-adrenal axis reactivity and regulation, as men with the GG genotype are particularly vulnerable to psychological stress, a crucial etiology for primary hypertension [30]. Taken together, these data suggest that higher central or circulating BDNF may account for hypertension and that the A variant may be linked to reduced BDNF levels. However, our findings are in accordance with those from a study of adults in South Korea that found that the *BDNF* G196A polymorphism significantly decreased systolic BP as a recessive SNP [31]. These findings can be interpreted as suggesting a protective role of the A allele in children via regulating BP and decreasing the risk of developing hypertension. However, there are studies with contradictory results; for example, Xi et al. reported that the *BDNF* G196A polymorphism was significantly associated with an increased risk of hypertension in Chinese children and adolescents [32]. Therefore, there is not yet enough evidence of the relationship between the *BDNF* G196A polymorphism and BP to draw a conclusion, and its mechanism remains to be elucidated, especially in children.

Notably, these inconsistent findings regarding the relationships between *BDNF* polymorphisms and obesity, blood pressure, and BDNF level among existing studies may be influenced by differences among populations in terms of ethnicity, lifestyle, severity of obesity, study design, and sample size. Moreover, a previous study has shown that bias might affect BDNF measurements, including sampling, handling, and storage procedures, leading to difficulties in interpreting the results [7]. However, in this study, blood was collected from children between 7.30 a.m. and 9.00 a.m. after a 12 h fast to minimize the effects of a possible circadian variation in circulating BDNF levels. Considering that platelets represent a major storage site of BDNF in peripheral blood [33], we measured plasma BDNF concentration because it represents the current steady state of biologically active BDNF, whereas serum concentration mainly reflects the content of platelets. Moreover, there was a difference between men and women in BDNF platelet content, but not plasma levels [34]. However, there are some limitations to our study. Firstly, the distribution of body fat and its visceral deposits were not directly assessed using computed tomography. Secondly, this study did not assess the relationships between obesity and diet, lifestyle, or family background. Thirdly, this study had a relatively small number of subjects for genotyping. Finally, we did not analyze the entire *BDNF* gene in relation to obesity. Further studies should aim to determine whether our findings can be replicated using larger sample sizes.

## 5. Conclusion

The present study in Thai children observed that the BDNF levels of obese children did not significantly differ from those of children with a normal weight and that BDNF levels were associated with cardiometabolic risk factors, including BP, the TyG index, and WBC count; the *BDNF* G196A polymorphism was associated with decreased BP. Therefore, the *BDNF* G196A polymorphism, which might play a beneficial role in controlling BP in Thai children, is premature. These findings will help elucidate the associations among plasma BDNF levels, risk factors for cardiovascular dysfunction, and *BDNF* polymorphisms in Thai children.

## Figures and Tables

**Table 1:** Comparison of the anthropometric-cardiometabolic and hematological variables between nonobese and obese children.

Variables	Nonobese group	Obese group	*p* value
	(*N* = 279)	(*N* = 190)	
Age (years)	10.6 ± 0.8	10.5 ± 0.9	0.138
Weight (kg)	36.0 ± 7.4	59.5 ± 10.5	<0.001^*∗∗*^
BMI (kg/m^2^)	18.8 ± 1.9	27.8 ± 3.1	<0.001^*∗∗*^
BMI z-score	0.1 (−0.6, 0.7)	2.9 (2.7, 3.2)	<0.001^*∗∗*^
WC (cm)	62.0 ± 7.6	86.4 ± 9.7	<0.001^*∗∗*^
Systolic BP (mmHg)	100.0 ± 13.4	113.0 ± 13.3	<0.001^*∗∗*^
Diastolic BP (mmHg)	63.2 ± 9.9	71.4 ± 10.1	<0.001^*∗∗*^
Glucose (mg/dL)	86.2 ± 6.5	88.7 ± 7.0	<0.001^*∗∗*^
TC (mg/dL)	187.6 ± 31.0	184.8 ± 31.9	0.327
TG (mg/dL)	60.0 (47.0, 79.0)	80.0 (60.0, 109.0)	<0.001^*∗∗*^
LDL-C (mg/dL)	118.0 ± 27.0	120 ± 28.0	0.193
HDL-C (mg/dL)	56.0 ± 11.0	46.0 ± 9.0	<0.001^*∗∗*^
Non-HDL-C (mg/dL)	131.0 ± 27.1	138.0 ± 28.7	<0.004^*∗∗*^
TyG index	8.0 ± 0.4	8.3 ± 0.4	<0.001^*∗∗*^
RBC count (×10^12^/L)	5.0 (4.7, 5.4)	5.0 (4.8, 5.3)	0.499
WBC count (×10^9^/L)	6.4 (5.4, 7.4)	8.1 (7.1, 9.7)	<0.001^*∗∗*^
Platelet count (×10^9^/L)	316.4 ± 57.3	320.0 ± 46.7	0.132
BDNF (ng/mL)	32.2 ± 6.4	33.8 ± 6.4	0.079

Data are means ± standard deviation or medians with interquartile range (25th–75th percentile); significance levels: ^*∗∗*^*p* < 0.01.

**Table 2:** Correlation coefficients of brain-derived neurotrophic factor concentration with other parameters in all subjects.

Variables	BDNF
Age	−0.029
Weight	0.010
BMI	0.086
BMI z-score	0.089
WC	0.091
Systolic BP	0.185^*∗∗*^
Diastolic BP	0.128^*∗*^
Glucose	0.101
TC	0.030
TG	0.158^*∗*^
HDL-C	−0.020
LDL-C	0.057
Non-HDL-C	0.029
TyG index	0.186^*∗∗*^
RBC count	0.198^*∗∗*^
WBC count	0.242^*∗∗*^
Platelet count	0.383^*∗∗*^

Significance levels: ^*∗*^*p* < 0.05, ^*∗∗*^*p* < 0.01.

**Table 3:** Logistic regression analysis of anthropometric-cardiometabolic and hematological variables associated with the single nucleotide polymorphisms *BDNF* G196A and C270T (*N* = 469).

Variables^†^	*BDNF* G196A	*p*-value	*BDNF* C270T	*p* value
	Odds ratio (95% CI)		Odds ratio (95% CI)	
Weight (kg)	0.990 (0.979–1.009)	0.145	1.011 (0.997–1.026)	0.132
BMI z-score	0.913 (0.885–1.025)	0.208	1.100 (0.959–1.262)	0.175
WC (cm)	0.992 (0.979–1.005)	0.224	1.011 (0.996–1.026)	0.149
Systolic BP (mmHg)	0.984 (0.971–0.998)	0.020^*∗*^	1.013 (0.998–1.030)	0.093
Diastolic BP (mmHg)	0.982 (0.963–1.000)	0.054	1.013 (0.992–1.033)	0.227
Glucose (mg/dL)	1.014 (0.985–1.025)	0.713	1.009 (0.975–1.043)	0.613
TC (mg/dL)	0.998 (0.991–1.008)	0.638	0.995 (0.988–1.002)	0.159
TG (mg/dL)	0.997 (0.991–1.005)	0.169	1.003 (0.997–1.009)	0.347
LDL-C (mg/dL)	1.009 (0.997–1.016)	0.232	0.997 (0.989–1.005)	0.501
HDL-C (mg/dL)	0.998 (0.981–1.015)	0.680	0.994 (0.989–1.005)	0.195
Non-HDL-C (mg/dL)	0.997 (0.991–1.007)	0.510	0.998 (0.990–1.006)	0.626
TyG index	0.680 (0.427–1.083)	0.104	1.405 (0.834–2.367)	0.201
RBC count (×10^12^/L)	0.822 (0.528–1.278)	0.383	0.923 (0.553–1.541)	0.761
WBC count (×10^9^/L)	0.998 (0.987–1.009)	0.724	0.995 (0.983–1.008)	0.471
Platelet count (×10^9^/L)	1.180 (0.827–1.686)	0.362	1.036 (0.697–1.540)	0.862
BDNF (ng/mL)	0.974 (0.941–1.008)	0.130	1.015 (0.980–1.022)	0.347

^†^Adjusted for the covariates age and gender, when SNP *BDNF* G196A (the absence vs. the presence of the A allele) or SNP *BDNF* C270T (the absence vs. the presence of the T allele) was used as a dependent variable. ^*∗*^*p* < 0.05.

**Table 4:** Genotypic and allelic distribution of the single nucleotide polymorphisms *BDNF* G196A and C270T in nonobese and obese children.

	Obese	Nonobese	Genotypic or allelic
	*N* (%)	*N* (%)	*p* value
*BDNF G196A*			
AA	40 (21.0)	61 (21.9)	
AG	82 (43.2)	137 (49.1)	0.284
GG	68 (35.8)	81 (29.0)	
GG vs. GA + AA^a^	0.721 (0.486–1.070)	Reference	
	[0.104]		
A allele	0.43 (162)	0.46 (259)	0.252
G allele	0.57 (218)	0.54 (299)	

*BDNF C270T*			
TT	2 (1.1)	3 (1.1)	
TC	43 (22.6)	55 (19.7)	0.747
CC	145 (76.3)	221 (79.2)	
CC vs. CT + TT^a^	1.183 (0.769–1.840)	Reference	
	[0.457]		
T allele	0.12 (47)	0.11 (61)	0.499
C allele	0.88 (333)	0.89 (497)	

^a^Values are the odds ratio with 95% confidence interval in parentheses; *p* values are in brackets.

**Table 5:** Logistic regression analysis with obesity used as a dependent variable and age, sex, brain-derived neurotrophic factor concentration, glucose, triglyceride-glucose (TyG) index, non-high-density lipoprotein cholesterol, white blood cell (WBC) count, the single nucleotide polymorphisms *BDNF* G196A, and C270T used as independent variables.

Variables	*β*	Odds ratios	95% CI	*p* value
Glucose	0.084	1.088	1.025–1.156	0.005^*∗∗*^
TyG index	1.136	3.113	1.294–5.488	0.009^*∗∗*^
WBC count	0.062	1.064	1.030–1.090	<0.001^*∗∗*^

95% CI is the 95% confidence interval of the odds ratio. Significance level: ^*∗∗*^*p* < 0.01.

## Data Availability

The data supporting the findings of this study are available within the article and supplementary materials. Raw data that support the findings of this study are available from the corresponding author upon reasonable request.
